# Molecular Diversity of Peptide Toxins in the Venom of Spider *Heteropoda pingtungensis* as Revealed by cDNA Library and Transcriptome Sequencing Analysis

**DOI:** 10.3390/toxins14020140

**Published:** 2022-02-14

**Authors:** Qingyi Liao, Xiangjin Kong, Guoqing Luo, Xiangyue Wu, Yinping Li, Qicai Liu, Cheng Tang, Zhonghua Liu

**Affiliations:** The National and Local Joint Engineering Laboratory of Animal Peptide Drug Development, College of Life Sciences, Hunan Normal University, Changsha 410081, China; qingyiliao@hunnu.edu.cn (Q.L.); kongxiangjin@hunnu.edu.cn (X.K.); lgq@hunnu.edu.cn (G.L.); wuxiangyue@hunnu.edu.cn (X.W.); liyinping@hunnu.edu.cn (Y.L.); qicailiu@hunnu.edu.cn (Q.L.)

**Keywords:** *Heteropoda pingtungensis*, cDNA library, transcriptome sequencing, peptide toxin, diversity

## Abstract

The venoms of toxic animals are chemical pools composed of various proteins, peptides, and small organic molecules used for predation and defense, in which the peptidic toxins have been intensively pursued mining modulators targeting disease-related ion channels and receptors as valuable drug pioneers. In the present study, we uncovered the molecular diversity of peptide toxins in the venom of the spider *Heteropoda pingtungensis (H. pingtungensis)* by using a combinatory strategy of venom gland cDNA library and transcriptome sequencing (RNA-seq). An amount of 991 high-quality expressed sequence tags (ESTs) were identified from 1138 generated sequences, which fall into three categories, such as the toxin-like ESTs (531, 53.58%), the cellular component ESTs (255, 25.73%), and the no-match ESTs (205, 20.69%), as determined by gene function annotations. Of them, 190 non-redundant toxin-like peptides were identified and can be artificially grouped into 13 families based on their sequence homology and cysteine frameworks (families A–M). The predicted mature toxins contain 2–10 cysteines, which are predicted to form intramolecular disulfide bonds to stabilize their three-dimensional structures. Bioinformatics analysis showed that toxins from *H. pingtungensis* venom have high sequences variability and the biological targets for most toxins are unpredictable due to lack of homology to toxins with known functions in the database. Furthermore, RP-HPLC and MALDI-TOF analyses have identified a total of 110 different peptides physically existing in the *H. pingtungensis* venom, and many RP-HPLC fractions showed potent inhibitory activity on the heterologously expressed NaV1.7 channel. Most importantly, two novel NaV1.7 peptide antagonists, µ-Sparatoxin-Hp1 and µ-Sparatoxin-Hp2, were characterized. In conclusion, the present study has added many new members to the spider toxin superfamily and built the foundation for identifying novel modulators targeting ion channels in the *H. pingtungensis* venom.

## 1. Introduction

Rather than strength, speed, and intelligence which make most predator species greatly adapted to the cruel “struggle for existence” on earth, toxic animals have evolved to utilize another smart and high-efficiency strategy, envenomation, for predation and defense [1]. At least 200,000 toxic animal species, including various snakes, scorpions, cone snails, centipedes, and spiders, were found all over the world [2]. Among them, more than 49,700 spiders are characterized (World Spider Catalog Version 22.5) to date, but few of their venoms, which are composed of cysteine-rich peptides, protein enzymes, and small organic molecules, are intensively studied. These peptidic toxins in spider venoms represent a valuable source for developing environment-friendly bioinsecticide, as most spiders predate by paralyzing or killing insects using their venom peptides to modulate the activity of insect ion channels, receptors, and enzymes [3,4]. On the other hand, these peptide toxins are also validated to modulate the activities of the various mammalian ion channels, which are the second most important class of drug targets after G-protein-coupled receptors, making them valuable and rich mines for drug development for treating channelopathies [5,6,7,8].

Spider peptide toxins are produced by the specialized venom gland. The secretory epithelial cells in it produce the toxin as a precursor, usually comprising the signal peptide, the propeptide, and the mature peptide, which will be cleaved to release the functional mature peptide during the exocytosis process [9]. These peptide toxins are small cysteine-rich proteins with a molecular weight of less than 10 kDa. Moreover, the cysteines in toxin form stable intramolecular disulfide bonds which assist it in refolding into a highly compacted globin-like structure, with the hydrophobic residues interacting to make a hydrophobic core and hydrophilic residues exposed to the aqueous phase. This structure makes the peptide toxins ultra-resistant to be destroyed by environmental challenges like heat, acid, base, and protein enzyme digestion. There are three commonly observed cysteine connecting modes in peptide toxins: the ICK(inhibitor cystine-knot) motif (disulfide mode: C_1_-C_4,_ C_2_-C_5_, C_3_-C_6_; the subscript number indicated the relative position of cysteine (C) in the sequence, the same hereinafter), the Kunitz motif (C_1_-C_6_, C_2_-C_4_, C_3_-C_5_), and the DDH(disulfide-directed β-hairpin) motif (C_1_-C_3_, C_2_-C_5_, C_4_-C_6_) [10,11]. It was estimated that each venom contains an average of at least 200 different peptides, which makes the potential peptide toxins in existing toxic animals astronomical [12]. However, the small body size and populations of toxic animals, as well as the small amount of venom produced by each animal, makes it hard to get enough venom from some species for further activity-guided purification and pharmacological analysis. Fortunately, cDNA library analysis of the venom gland has provided an alternative strategy to investigate the diversity of peptide toxins at the cost of only several venom glands from the animals. Moreover, high throughput transcriptome sequencing can quickly uncover the whole transcriptome of the venom gland without the need for cDNA library construction and time-consuming clone sequencing, although it does not bias the sequencing of peptide toxin transcripts. The information of venom peptide sequences can greatly facilitate our identification of drug pioneers and molecular probes targeting ion channels as (1) with the fast growth of ion channel structures in the PDB database and a deeper understanding of the mechanisms of peptide-channel interactions, it is becoming practical for in silico screening of channels’ modulators based on modeled peptide toxins by knowing their sequences; (2) several talent strategies by using the peptide sequences in the library, such as phage-displayed toxins and tethered-toxins screening, have been developed in recent years and proved to be powerful for fishing peptidic modulators targeting the ion channels without producing the recombinant toxins [13,14]. 

*Heteropoda pingtungensis (H. pingtungensis)* is a spider species belonging to the *Heteropodidae* family, characterized in 2006, in Taiwan, China (https://wsc.nmbe.ch/species/34674/Heteropoda_pingtungensis; accessed on 23 January 2022) [15]. It is also distributed in the Yunnan, Guizhou, Guangdong, and Guangxi provinces of China. Up to date, more than 1000 spider species are identified in the *Heteropodidae* family and the venom peptides from one member, the *Heteropoda venatoria (H. venatoria)*, are intensively explored by both venom fractionation-based active peptides screening and venom gland cDNA library sequencing [11,16,17,18,19]. However, the venom components of *H. pingtungensis* and their biological activities are never explored. In the present study, we revealed the great diversity of *H. pingtungensis* venom peptides using a combinatory strategy of cDNA library and transcriptome sequencing, from which 190 novel toxin-like peptides belonging to 13 families are identified. In line with this, RP-HPLC fractionation and MALDI-TOF analysis revealed approximately 110 different peptide toxins existing in the venom. Additionally, electrophysiology analysis identified several RP-HPLC purified fractions of *H. pingtungensis* venom with potent inhibitory activity on the pain-related NaV1.7 channel, showing the venom as a valuable source for active peptides screening. In summary, the present study has expanded the capacity of the existing spider toxins’ superfamily and set the foundation for identifying novel modulators targeting ion channels and receptors from the *H. pingtungensis* venom. 

## 2. Results and Discussion

### 2.1. General Features and Annotations of H. Pingtungensis Venom Gland ESTs

**Figure 1:**
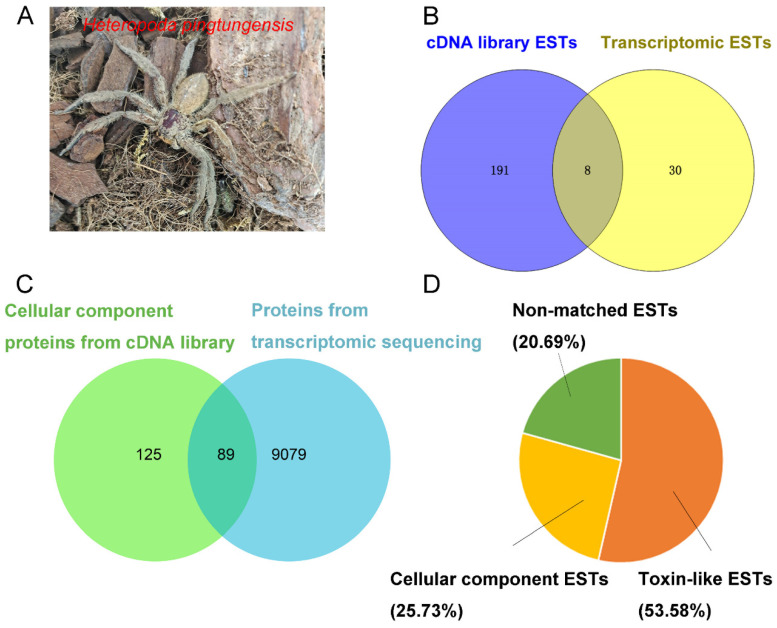
*H. pingtungensis* venom gland ESTs annotations and distributions. (**A**) The spider *H. pingtungensis*. (**B**) Toxin-like ESTs derived from cDNA library and/or transcriptome sequencing. (**C**) Overlap of cellular component proteins identified in the cDNA library group with those proteins derived from transcriptome sequencing. (**D**) The relative proportion of each transcript category of *H. pingtungensis* venom gland ESTs.

Clone sequencing of the *H. pingtungensis* (Figure 1A) venom gland in the cDNA library resulted in 953 high-quality ESTs, which are grouped into three distinct functional clusters by blasting against the non-redundant protein sequence (nr) database: 493 ESTs encode putative toxins (toxin-like ESTs), with the cDNA length ranging from 0.3–1.0 Kb (average of 560 bp); 255 ESTs are other cellular protein-encoding sequences (cellular component ESTs), with the cDNA length ranging from 0.45–1.0 Kb (average of 733 bp); and 205 ESTs do not match any sequences in the database (non-matched ESTs), with the cDNA length ranging from 0.2–1.05 Kb (average of 753 bp). These data suggested that the toxin-like ESTs are greatly enriched in the cDNA library. On the other hand, 38 toxin-like ESTs are extracted from the transcriptomic data, and their length ranges from 0.15–0.45 Kb (average of 240 bp). The toxin-like ESTs from cDNA library sequencing are rarely overlapped with those from transcriptomic sequencing, suggesting the two strategies are complementary in discovering the peptide toxin genes (Figure 1B). Moreover, significantly more, longer toxin-like ESTs are identified from cDNA library sequencing, suggesting that transcriptome sequencing is limited in revealing the whole landscape of peptide toxin transcripts in the venom gland without an enrichment strategy. Blasting the cellular component proteins identified by cDNA library sequencing against the protein sequence database built by transcriptome sequencing showed that 89 proteins in the cDNA library group have a 100% identity with proteins in the transcriptomic group, accounting for 41.5% of total cellular component proteins identified in the former (Figure 1C). It should be noted that the sequence of cellular component proteins is much longer than that of toxin-like peptides, and these two approaches may identify different regions of the same protein, therefore such blast analysis may underestimate the overlap between these two groups. Nonetheless, this overlap proportion is significantly higher than that in the toxin-like group (4.02%). Taken together, out of a total of 991 high-quality ESTs, 531 ESTs (53.58%) encode the putative toxin precursors, 255 ESTs (25.73%) encode other cellular proteins, and 205 ESTs (20.69%) do not hit any known proteins in the database (Figure 1D).

### 2.2. Cluster Analysis of H. Pingtungensis Venom Gland ESTs

**Figure 2:**
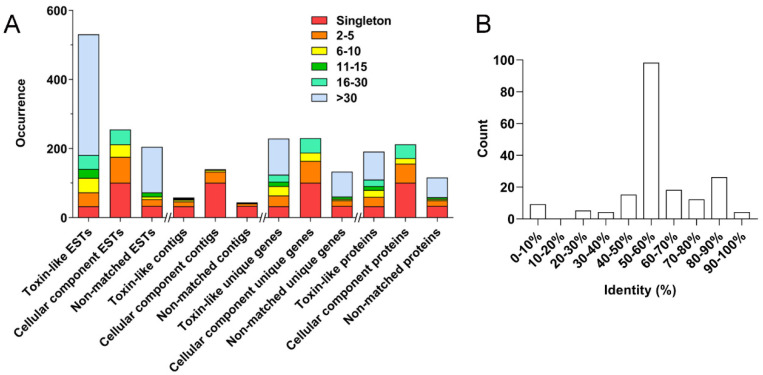
(**A**) EST, contig, unique gene, and protein distributions of toxin-like, cellular component, and non-matched ESTs among different cluster groups. (**B**) Toxins from *H. pingtungensis* were blasted against the toxins sequence library from *H. venatoria*, and the number of toxins showing different levels of homology (0–100% identity, in 10% increment) was counted, showing the toxin precursors from these two close spider species are basically different.

As shown in Figure 2A, cluster analysis of the 991 ESTs results in 242 clusters, including 168 singletons and 74 contigs of different sizes (2–5, 6–10, 11–15, 16–30, and >30, which indicates the number of clustered sequences in each contig). Firstly, 531 toxin-like ESTs are grouped into 58 clusters, including 33 singletons and 25 contigs: (1) 14 contigs of size 2–5 contain 40 ESTs, which represent 31 unique genes encoding 27 proteins; (2) 5 contigs of size 6–10 contain 42 ESTs, which represent 27 unique genes encoding 20 proteins; (3) 2 contigs of size 11–15 contain 26 ESTs, which represent 13 unique genes encoding 11 proteins; (4) 2 contigs of size 16–30 contain 40 ESTs, which represent 20 unique genes encoding 19 proteins; (5) 2 contig of size >30 contains 350 ESTs, which represent 105 unique genes encoding 80 proteins. 

Secondly, the cellular component ESTs are grouped into 140 clusters, including 101 singletons and 39 contigs: (1) 32 contigs of size 2–5 contain 75 ESTs, which represent 63 unique genes encoding 55 proteins; (2) 5 contigs of size 6–10 contain 36 ESTs, which represent 24 unique genes encoding 16 proteins; (3) 2 contigs of size 16–30 contain 43 ESTs, which represent 42 unique genes encoding 40 proteins. No contigs of size 11–15 and >30 are observed in this category.

Lastly, the non-matched ESTs are grouped into 44 clusters, including 34 singletons and 10 contigs: (1) 7 contigs of size 2–5 contain 19 ESTs, which represent 16 unique genes encoding 16 proteins; (2) 1 contig of size 6–10 contain 8 ESTs, which represent 4 unique genes encoding 4 proteins; (3) 1 contig of size 11–15 contain 12 ESTs, which represent 7 unique genes encoding 5 proteins; (4) 1 contig of size > 30 contain 132 ESTs, which represent 72 unique genes encoding 57 proteins. No contigs of size 16–30 are presented in this category.

From the above analysis, one can find that most toxin-like ESTs (93.79%) are grouped into contigs, which are consistent with the notion that most peptide toxin transcripts are always high-copy in the venom gland. The high-abundance ESTs in the cellular component category, however, mostly encode ribosomal proteins, which is also in line with the active protein synthesis in the venom gland. Accordingly, a total of 518 putative non-redundant proteins were identified in the *H. pingtungensis* venom gland, including 190 toxin precursors, 212 cellular component proteins, and 116 non-matched proteins. With BLAST, all these toxin precursors from *H. pingtungensis* against the toxins sequence database from the close spider species, *H. venatoria*, showed that their toxins are generally of medium homology, with most of the toxins (98 out of a total of 190) from *H. pingtungensis* showing 50–60% sequence identity with those from *H. venatoria* (Figure 2B).

### 2.3. Family Analysis of Putative Toxin Precursors in H. Pingtungensis Venom Gland

**Figure 3:**
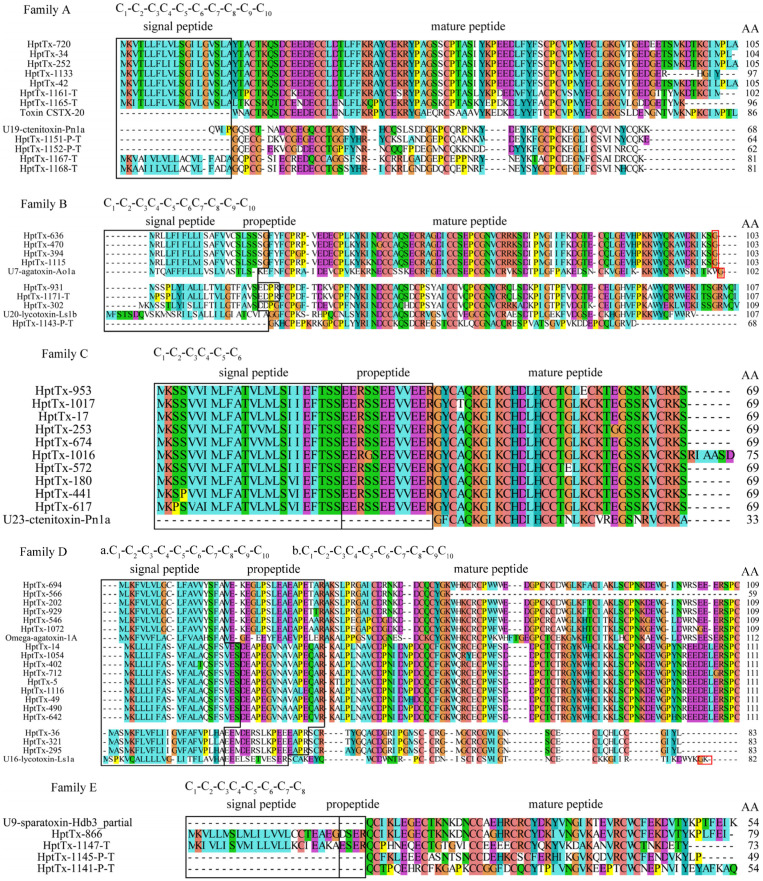

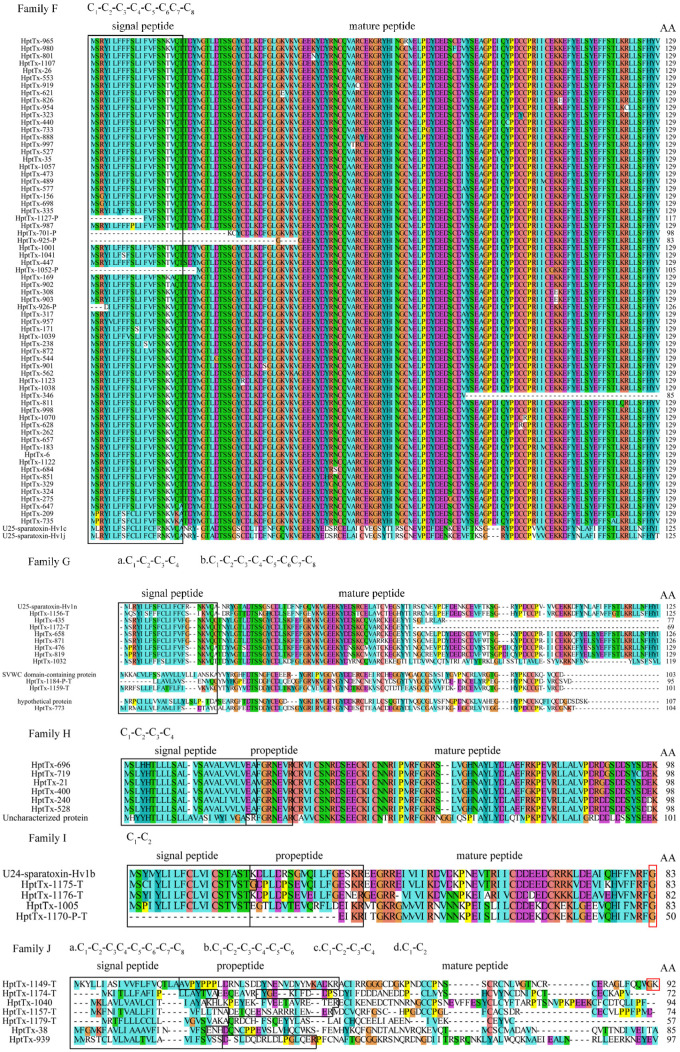

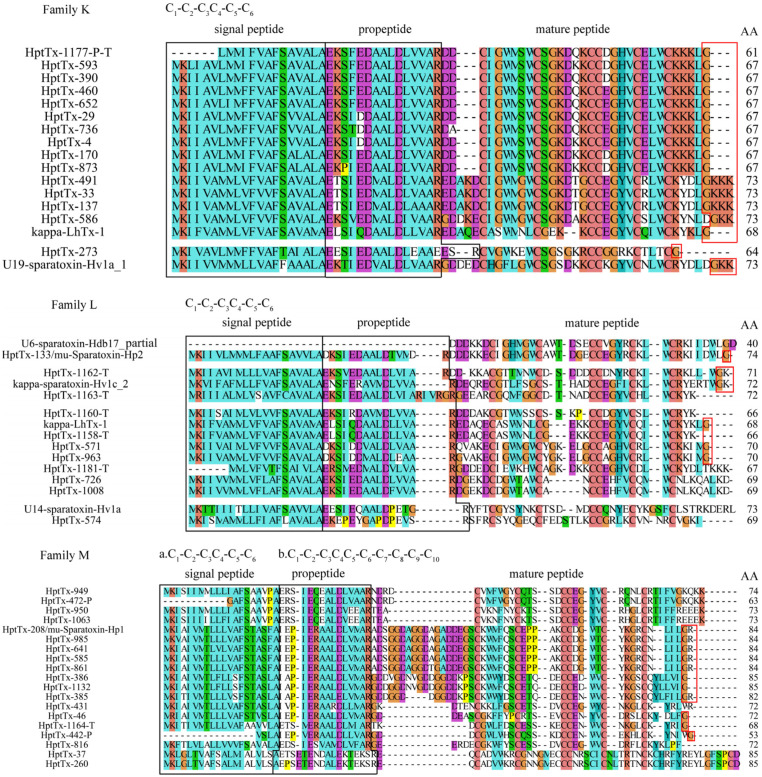
Family analysis of toxin-like precursors in the venom gland of *H. pingtungensis*. The black-boxed regions indicate signal peptides and possible propeptides, and the red-boxed regions indicate the C-terminal amidation signals.

We named the toxin-like peptide from *H. pingtungensis* as HptTx-n, in which ‘Hpt’, ‘Tx’, and ‘n’ represents *H. pingtungensis*, toxin, and the clone number, respectively. In some cases, the capital letter ‘P’ or ‘T’ is added to the end of the toxin name to indicate that toxin is a partial sequence (HptTx-n-P) or derived from transcriptomic data (HptTx-n-T), respectively. BLAST analysis revealed that 176 out of 190 toxin precursors are likely of full-length with the complete signal peptide and mature peptide, for which the signal peptide was predicted using signalP5.0 (https://services.healthtech.dtu.dk/service.php?SignalP-5.0; accessed on 23 January 2022) [20], and the cut site between the propeptide and predicted mature peptide was determined by the Processing Quadruplet Motif (PQM) mode [21,22]. Sequences of all these novel toxins were deposited in the Genbank database (https://www.ncbi.nlm.nih.gov/genbank/; accessed on 24 January 2022) (Genbank accession numbers: OM362623—OM362812). As shown in Figure 3, these toxins were grouped into 13 families based on their sequence homology and cysteine frameworks. 

#### 2.3.1. Family A

Family A contains 11 precursor toxins, all of which lack a typical propeptide cutting site. (1) HptTx-720, HptTx-34, HptTx-252, HptTx-1133, HptTx-42, HptTx-1161-T, and HptTx-1165-T are a subset of toxins showing the highest homology with toxin CSTX-20 (64–70% identity) from the spider *Cupiennius* salei (*American wandering spider*) and U4-sparatoxin-Hv1a (75–91% identity) from the spider *Heteropoda venatoria*, when blasted against the Uniprot and NR databases, respectively. Both these two hit toxins’ biological functions are unknown. HpTx-1161-T and HpTx-1165-T are derived from transcriptome sequencing and thus might represent the artificially C-terminus truncated peptides. Interestingly, HptTx-1133 lacks the most C-terminal cysteine residue, whereas its cDNA contains a complete 3′ poly-A tail, suggesting it is a full-length peptide with its last cysteine residue mutated. (2) HptTx-1151-P-T, HptTx-1152-P-T, HptTx-1167-T, and HptTx-1168-T are derived from transcriptomic data and make another cluster of toxins in this family. Their predicted mature peptides showed the highest homology with U19-ctenitoxin-Pn1a (54–70% identity) from the spider *Phoneutria nigriventer* (*Brazilian armed spider*), which is non-toxic to mice and insects. HptTx-1167-T and HptTx-1168-T contain the complete signal peptide, whereas HptTx-1151-P-T and HptTx-1152-P-T are likely just the mature peptides. It is unknown whether their mature peptides are complete. All the predicted mature peptides for toxins in this family share a consensus cysteine framework as C_1_-C_2_-C_3_C_4_-C_5_-C_6_-C_7_-C_8_-C_9_-C_10_. This cysteine pattern is also seen in the snake toxin, Toxin MIT1, in which the cysteine connecting mode is determined as C_1-4_, C_2-5_, C_3-7_, C_6-9_, and C_8-10_ [23]. We supposed that these toxins have the same disulfide mode as Toxin MIT1. 

#### 2.3.2. Family B

Family B contains eight precursor sequences. Except for HptTx-1143-P-T, which is derived from transcriptome sequencing and has the lowest homology with other toxins in this family, all the other eight toxins are of full-length. HptTx-931, HptTx-1171-T, and HptTx-302 cluster into a subset showing the highest homology with U20-lycotoxin-Ls1b (51–52% identity) from the spider *Lycosa Singoriensis* and U6-sparatoxin-Hv1h (80–84% identity) from the spider *Heteropoda venatoria*, in the Uniprot and NR databases, respectively. The former was predicted to have antibacterial activity. Compared with HptTx-931 and HptTx-1171-T, HptTx-302 is supposed to lack the propeptide due to an R to G mutation at the PQM region. HptTx-636, HptTx-470, HptTx-394, and HptTx-1115 cluster into another group, with the signal peptide cutting site being determined as SSG/FY. No propetide is presented in their sequences. Blasting analysis showed they share the highest homology with U7-agatoxin-Ao1a from the spider *Agelena orientalis* (52–53% identity, Uniprot database) and U7-sparatoxin-Hv1a_1 from the spider *Heteropoda venatoria* (87–88% identity, NR database), both of whose biological functions are unknown. Predicted mature peptides for toxin precursors in this family have a consensus cysteine framework as C_1_-C_2_-C_3_C_4_-C_5_-C_6_C_7_-C_8_-C_9_-C_10_, and the putative disulfide mode is C_1_-C_7_, C_2_-C_8_, C_3_-C_6_, C_4_-C_10_, and C_5_-C_9_ based on similarity analysis.

#### 2.3.3. Family C

The ten peptide precursors in family C showed great homology with each other, with a variation of residues being only present in the signal peptide and/or propeptide regions in several members. All of them are from cDNA library sequencing and are of full-length. The predicted mature peptides of this family are best aligned to the toxin U23-ctenitoxin-Pn1a from the spider *Phoneutria nigriventer* (73–76% identity, Uniprot database) and U22-sparatoxin-Hv1a from the spider *Heteropoda venatoria* (79–82% identity, NR database). Both these two toxins’ biological functions are unknown, but the former was shown to be non-toxic to mice. Interestingly, HptTx-1016 has a longer mature peptide than the others, which might be caused by a single nucleotide mutation in the stop codon, as revealed by analyzing their cDNA sequences (the first stop codon TGA in several other toxins is mutated to CGA in HptTx-1016). It is unknown whether such a mature peptide extension would render new function to this toxin. Furthermore, their classic cysteine framework, C_1_-C_2_-C_3_C_4_-C_5_-C_6_, as observed in most inhibitor cysteine knot (ICK) motif spider toxins, strongly suggests the mature peptides in this family are also knotting toxins (disulfide mode: C_1_-C_4_, C_2_-C_5_, and C_3_-C_6_).

#### 2.3.4. Family D

There are three clusters of peptide precursors in this family, which all contain a signal peptide and a propeptide. Among them, the first cluster of toxins (HptTx-694, HptTx-566, HptTx-202, HptTx-929, HptTx-546, and HptTx-1072) match the best with toxin omega-agatoxin-1A from the spider *Agelenopsis aperta* in the Uniprot database (58–65% identity and 71–75% similarity). This toxin is previously determined to be a blocker of presynaptic calcium channels at the insect neuromuscular junction [24,25]. The high sequence homology of their mature peptides with that of omega-agatoxin-1A suggests that they may share the same biological targets and play a key role for *H. pingtungensis* to prey on insects. The second group of toxins, including HptTx-14, HptTx-1054, HptTx-402, HptTx-712, HptTx-5, HptTx-1116, HptTx-49, HptTx-490, and HptTx-642, are also best aligned with omega-agatoxin-1A but with lower homology than the first cluster of toxins (47–49% identity and 57–59% similarity). Both these two classes of toxins have a consensus cysteine framework as C_1_-C_2_-C_3_-C_4_-C_5_-C_6_-C_7_-C_8_-C_9_-C_10_, the same as omega-agatoxin-1A. The other three toxins in this family (HptTx-36, HptTx-321, and HptTx-295), however, have another different cysteine framework with their last two cysteines directly connected (C_1_-C_2_-C_3_-C_4_-C_5_-C_6_-C_7_-C_8_-C_9_C_10_). Moreover, blasting analysis revealed that they are best aligned to, although with relatively low homology, U16-lycotoxin-Ls1a (35–36% identity), which was predicted to have ion channel inhibitor activity but is currently not experimentally verified.

#### 2.3.5. Family E

Family E contains four toxin precursors, with HptTx-866 being derived from the cDNA library and the other three from transcriptome sequencing. Consequently, it remains elusive whether the mature peptides in the latter three toxins are complete. Among them, HptTx-1147-T has the signal peptide and propeptide, whereas HptTx-1145-P-T and HptTx-1141-P-T seem to be just the mature peptides. The overall sequence homology between these four toxins is relatively low but they share a consensus cysteine framework as C_1_-C_2_-C_3_C_4_-C_5_-C_6_-C_7_-C_8_. Blasting analysis revealed they all have moderate sequence homology with U6-lycotoxin-Ls1g from the spider *Lycosa singoriensis* (40–54% identity, Uniprot database), which has an unknown biological function. Moreover, blasting against the NR database showed that HptTx-1141-P-T has the same mature peptide as U9-sparatoxin-Hdb4, partial (100% identity), and HptTx-866 has extremely high homology with U9-sparatoxin-Hdb3, partial (91% identity), from the spider *Heteropoda davidbowie*. 

#### 2.3.6. Family F

There are 66 toxin precursors in family F, which represents the most abundant cluster in the cDNA library. Five toxins (HptTx-1127-P, HptTx-701-P, HptTx-925-P, HptTx-926-P, and HptTx-1052-P) in this family with a truncated N-terminus should be caused by mRNA fragmentation during cDNA library construction, whereas the toxin HptTx-346 with a truncated C-terminus is generated by a tyrosine codon (UAC) mutation to the stop codon (UAG). These toxins are of extremely high homology and are made of short signal peptides and rather long predicted mature peptides, which have a consensus cysteine framework of C_1_-C_2_-C_3_-C_4_-C_5_-C_6_C_7_-C_8_. However, there are some toxins with mutations at the conserved cysteine sites, including HptTx-980, HptTx-965, HptTx-1123, HptTx-323, HptTx-628, HptTx-888, HptTx-925-P, HptTx-1052-P, and HptTx-1070. On the other hand, HptTx-701-P, HptTx-440, HptTx-919, and HptTx-954 have an additional cysteine in their sequences except for the common cysteine framework. Blasting analysis of their sequences against the UniProt database results in no significant hits, which makes it impossible to predict their biological functions. However, they showed moderate homology with U25-sparatoxin-Hv1c and U25-sparatoxin-Hv1j from the spider *Heteropoda venatoria* in the NR database (58–62% identity and 75–80% similarity). Interestingly, the transcripts for these two hit toxins are also the top two most abundant in the venom cDNA library of *H. Venatoria* [11], raising the possibility that this class of toxins plays an important function in prey and defense for these two spider species.

#### 2.3.7. Family G

The eleven toxin precursors in this family can be divided into three groups, with no typical propeptide cutting sites found in these toxins. HptTx-1156-T, HptTx-658, HptTx-476, and HptTx-819 have a consensus cysteine framework of C_1_-C_2_-C_3_-C_4_-C_5_-C_6_C_7_-C_8_, as those toxins in family F. HptTx-871 have high sequence homology with HP-658 but its third cysteine (C_3_) is mutated to arginine. The sequence of HptTx-435 is much shorter than the others because of a premature termination codon in its sequence, and HptTx-1172-T derived from the transcriptomic data might represent an artificial C-terminus truncated peptide. These toxins showed the highest homology with U25-sparatoxin-Hv1n from the spider *Heteropoda venatoria* (67–71% identity). Unlike other U25-sparatoxin-Hv1n related peptides in this subset, HptTx-1032 has a different cysteine framework of C_1_-C_2_-C_3_-C_4_. HptTx-773, HptTx-1184-P-T, and HptTx-1159-T also have a cysteine pattern of C_1_-C_2_-C_3_-C_4_-C_5_-C_6_C_7_-C_8,_ with HptTx-773 being best aligned with hypothetical protein AVEN_267295-1 from spider *Araneus ventricosus* (44% identity), and the latter two toxins have moderate sequence homology with the SVWC domain-containing protein from the spider *Caerostris darwini* (42–45% identity). Furthermore, the predicted mature peptides in HptTx-1184-P-T and HptTx-1159-T might not be complete. We are unable to predict the functions of toxins in family G based on sequence homology analysis.

#### 2.3.8. Family H

There are six toxin precursor peptides in this family, all of which are derived from cDNA libraries. Except for HptTx-719, which has an extra cysteine residue at its C-terminus, all other toxins in this family have a cysteine pattern of C_1_-C_2_-C_3_-C_4_. A short propeptide is presented between their signal and the predicted mature peptides. The overall sequence homology between family H members is extremely high, and blasting analysis showed they are best aligned with uncharacterized protein TNCT_234811 from the spider *Trichonephila clavate* (55–57% identity and 70–71% similarity, NR database)*,* which has an unknown biological function. 

#### 2.3.9. Family I

This family has four toxin precursor sequences, whose predicted mature peptides have only two cysteines (cysteine framework: C_1_-C_2_). HptTx-1005 is derived from cDNA library sequencing, and HptTx-1175-T, HptTx-1176-T, and HptTx-1170-P-T are from transcriptomic data. These four toxin precursors have completed mature peptides, with an amidation signal at their C-termini (the C-terminal glycine residue), which would result in amidation of its upstream phenylalanine residue at the carboxyl group. All these toxins are bested aligned with U24-sparatoxin-Hv1b from the spider *Heteropoda venatoria* (52–82% identity, NR database), which has an unknown biological function. Interestingly, the predicted mature toxins from family I members all have an -RFamide sequence at their C-termini, which is a characteristic feature of FMRFamide-related peptides (FaRPs), therefore, these toxins are also likely to activate respective GPCRs(G-protein-coupled receptors). Actually, FaRPs are also identified in other toxic animals, such as the solitary wasp and cone snail [26,27].

#### 2.3.10. Family J

Family J contains seven toxin precursors that are of very low sequence homology. Among them, HptTx-1149-T, HptTx-1174-T, HptTx-1157-T, and HptTx-1179-T are derived from transcriptomic data. HptTx-1149-T has a complete mature peptide, as revealed by a typical amidation motif (GK) at its C-terminus, whereas the mature peptide of HptTx-1179-T might be incomplete, as only five cysteines are presented in its sequence. It is unknown whether HptTx-1174-T and HptTx-1157-T have complete mature peptides. (1) HptTx-1149-T matches the best with the U8-agatoxin-Ao1a toxin from the spider *Stegodyphus dumicola* (37% identity) and U8-agatoxin-Ao1a-like toxin from the spider *Stegodyphus dumicola* (76% identity), in the Uniprot and NR database, respectively. (2) HptTx-1174-T has a cysteine pattern of C_1_-C_2_-C_3_-C_4_-C_5_-C_6_. Blasting analysis revealed its high homology with U1-sparatoxin-Hv1a from the spider *Heteropoda venatoria* (77% identity and 82% similarity). (3) HptTx-1040 is best aligned with U1-lycotoxin-Ls1b (33.7% identity, Uniprot database) from the spider *Lycosa singoriensis* and U3-sparatoxin-Hdb1, partial from the spider *Heteropoda davidbowie* (81% identity, NR database). (4) HptTx-1157-T is best aligned with U29-sparatoxin-Hv1a (65% identity, NR database) from the spider *Heteropoda venatoria*. Notably, HptTx-1149-T, HptTx-1040, and HptTx-1157-T share a consensus cysteine framework as C_1_-C_2_-C_3_C_4_-C_5_-C_6_-C_7_-C_8_, which is also a typical pattern in ICK motif toxins. The disulfide mode in them is speculated to be C_1-4_, C_2-5_, C_3-8_, C_6-7_. (5) HptTx-1179-T and HptTx-38 have no propeptides in their sequences. Blasting analysis showed HptTx-1179-T has the highest homology with U27-sparatoxin-Hv1c (79% identity) from the spider *Heteropoda venatoria*. (6) HptTx-38 has a cysteine framework of C_1_-C_2_-C_3_-C_4_ and is best aligned with hypothetical protein X975_08641, partial from the spider *Stegodyphus mimosarum* (64% identity). (7) HptTx-939 only has two cysteines in sequence (C_1_-C_2_). Blasting analysis showed it matches uncharacterized protein TNCT_72511 from the spider *Trichonephila clavate* (66% identity). We are unable to predict the biological functions of these toxins in family J as the functions of all their hit toxins in the database are unknown.

#### 2.3.11. Family K

There are 15 toxin precursors in family K, all of which have a propeptide in their sequences. Their predicted mature peptides share a consensus cysteine framework popularly found in ICK motif toxins (C_1_-C_2_-C_3_C_4_-C_5_-C_6_), suggesting the disulfide mode is C1-C_4_, C_2_-C_5_, and C_3_-C_6_. The C-terminal amidation signals are observed in family K members (red-boxed region), which implies the C-termini of these toxins are amidated when expressed in the venom. These toxins can be grouped into three subsets based on their sequence homology: cluster 1 (HptTx-1177-P, HptTx-460, HptTx-652, HptTx-390, HptTx-29, HptTx-4, HptTx-736, HptTx-170, HptTx-873, and HptTx-593), cluster 2 (HptTx-491, HptTx-33, HptTx-137, and HptTx-586), and cluster 3 (HptTx-273 alone). Blasting analysis revealed that most toxins, except for HptTx-273, in this family are best aligned with kappa-LhTx-1 from the spider *Pandercetes sp,* with cluster 2 toxins showing slightly higher sequence homology (65–70% identity and 75–80% similarity) than that of cluster 1 toxins (57–61% identity and 72–81% similarity). This spider toxin was proved in our previous study to be a Kv4 channels’ specific antagonist, which inhibits Kv4.1-4.3 subtypes with different voltage dependence [28]. HptTx-273, however, has the highest homology with U19-sparatoxin-Hv1a from the spider *Heteropoda venatoria* (57% identity and 69% similarity), whose biological function is not experimentally determined yet.

#### 2.3.12. Family L

This family has 11 toxin precursors, all of which have a propeptide and contain 6 cysteines arranged as C_1_-C_2_-C_3_C_4_-C_5_-C_6_. Based on this cysteine scaffold, typically observed in ICK motif toxins, their disulfide bond was predicted to be C_1-4_, C_2-5_, and C_3-6_. Toxins in this family are diverse and are divided into four subsets: (1) HptTx-571, HptTx-963, HptTx-1181-T, HptTx-1158-T, HptTx-726, HptTx-1008, and HptTx-1160-T are best aligned with kappa-LhTx-1 from the spider *Pandercetes* sp. Among them, HptTx-1158-T showed 100% sequence identity with kappa-LhTx-1 but its mature peptide is one residue shorter than the latter, suggesting HptTx-1158-T is not a complete sequence. The other toxins have moderate homology with kappa-LhTx-1 (59–61% identity and 71–78% similarity). HptTx-571 and HptTx-963 are homologous peptides with an amidation signal (the C-terminal glycine residue) at their C-termini, and HptTx-726 and HptTx-1008 only differ from each other by a single residue in the propeptide region. (2) HptTx-1162-T and HptTx-1163-T are derived from the transcriptomic data, and the mature peptide of HptTx-1162-T should be complete due to an amidation signal (GK) at its C-terminus. BLAST analysis showed that they have the highest homology with Kappa-sparatoxin-Hv1c_2 (56% identity and 72% similarity) and Kappa-sparatoxin-Hv1e_2 (56% identity and 59% similarity), from the spider *Heteropoda venatoria*, respectively. (3) HptTx-133 has an amidation signal at its C-terminus. Its predicted mature peptide has extremely high homology with the toxin U6-sparatoxin-Hdb17, partial from the spider *Heteropoda davidbowie* (92% identity and 94% similarity; (4) HptTx-574 is best aligned with U14-sparatoxin-Hv1a from the spider *Heteropoda venatoria* (42% identity and 55% similarity). It is worth noting that most toxins in family K and L are related to kappa-LhTx-1, which raises the possibility that they also act on Kv channels.

#### 2.3.13. Family M

This family has 19 precursor peptides, all of which contain propeptides. Except for HptTx-37 and HptTx-260 which have a novel cysteine framework of C_1_-C_2_-C_3_C_4_C_5_-C_6_-C_7_-C_8_-C_9_-C_10_, the other members are likely ICK motif toxins that have a consensus cysteines arrangement of C_1_-C_2_-C_3_C_4_-C_5_-C_6_, and the disulfide mode should be C_1-4_, C_2-5_, and C_3-6_. These toxins are relatively diverse and can be grouped as follows: (1) HptTx-949 and HptTx-472-P have the same predicted mature peptide and propeptide, but possibly different signal peptides. HptTx-950 and HptTx-1063 also differ from each other by only two residues in their signal peptide region. These four toxins are best aligned with μ-sparatoxin-Hv2 from the spider *Heteropoda venatoria* (43–46% identity and 62–65% similarity) and U20-sparatoxin-Hv1g from the spider *Heteropoda venatoria* (52–60% identity and 65–68% similarity), in the Uniprot and NR database, respectively. In our previous study, μ-sparatoxin-Hv2 was identified as an insecticidal toxin functioning by potently and irreversibly inhibiting the insect voltage-gated sodium channel [19], whereas the biological function of U20-sparatoxin-Hv1g is unknown; (2) HptTx-208, HptTx-985, HptTx-641, HptTx-585, and HptTx-861 are another homologous subset, and their predicted mature peptides are likely amidated at the C-terminus, as indicated by a GR amidation signal. BLAST analysis showed they are best aligned with μ-sparatoxin-Hv2 (40–41% identity and 52% similarity, Uniprot database); (3) HptTx-386, HptTx-1132, and HptTx-385 have high sequence homology with each other, their predicted mature peptides are also presumably amidated. These three peptides also have the highest homology with μ-sparatoxin-Hv2 (42–43% identity and 54–56% similarity, Uniprot database); (4) HptTx-431, HptTx-46, HptTx-1164-T, HptTx-442-P, and HptTx-816 have higher sequence homology with μ-sparatoxin-Hv2 than other toxins in this family (51–58% identity and 58–69% similarity). Except for HptTx-431 and HptTx-816, the predicted mature peptides of the other three toxins are likely amidated; (5) HptTx-37 and HP-260 differ by a residue in the propeptide region. They are best aligned with Kappa-ctenitoxin-Pn1a from the spider *Phoneutria nigriventer* in the Uniprot database, although with low sequence homology (35–36% identity and 51–53% similarity). However, they have high homology with U8-sparatoxin-Hdb1, partial from the spider *Heteropoda davidbowie* (73% identity and 84% similarity) in the NR database. 

### 2.4. RP-HPLC Profile of H. Pingtungensis Venom and Screening of NaV1.7 Inhibitory Peptides 

**Figure 4:**
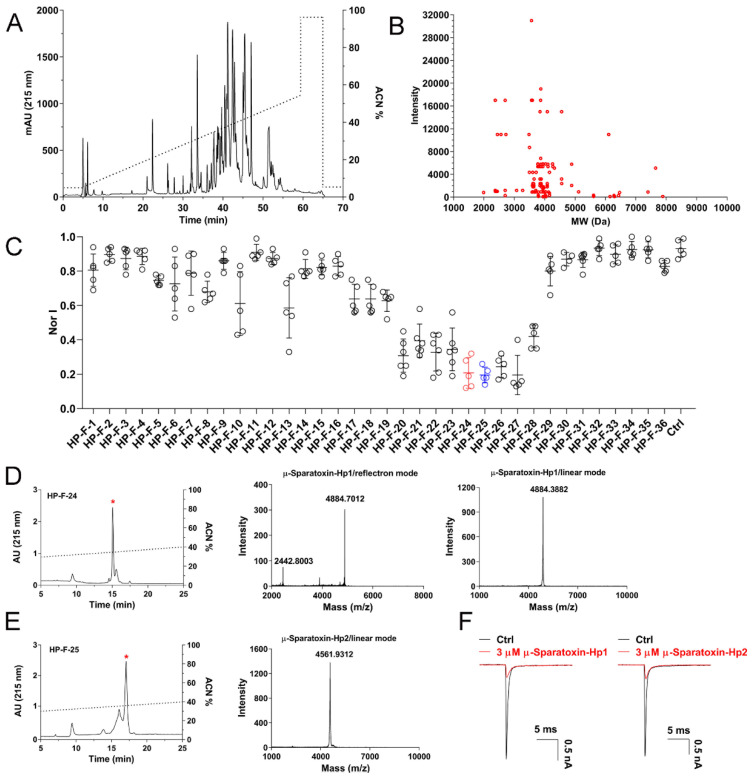
Venom peptides diversity in *H. pingtungensis* and NaV1.7 inhibition activity of venom fractions. (**A**) RP-HPLC profile of *H. pingtungensis* venom. (**B**) MALDI-TOF analysis showing the molecular weight (MW) distribution and intensity of venom peptides in *H. pingtungensis*. (**C**) NaV1.7 inhibition activity of each RP-HPLC fraction in (**A**), the final testing concentration of venom peptides in each fraction is approximately 2–3 µM. Each dot represents a separate experimental cell, data are presented as MEAN ± SD. (**D**) Analytical RP-HPLC purification of the fraction HP-F-24 (left; star labeled peak indicates µ-Sparatoxin-Hp1), and reflectron mode (middle) and linear mode MALDI-TOF MS (right) analysis of µ-Sparatoxin-Hp1. Note the MW of 2442.8003 in reflectron mode MS analysis represents the toxin carrying two H^+^ [(M + 2H^+^)/2], and the small peak with MW of 3888.6421 might be a toxin fragment, as it is not presented in linear mode MS analysis. (**E**) Analytical RP-HPLC purification of the fraction HP-F-25 (left; star labeled peak indicates µ-Sparatoxin-Hp2) and linear mode MALDI-TOF MS (right) analysis of µ-Sparatoxin-Hp2. The toxin was not detected by reflectron mode MS analysis. (**F**) Representative traces showing the inhibition of 3 µM µ-Sparatoxin-Hp1 and µ-Sparatoxin-Hp2 on NaV1.7 currents (*n* = 5–6).

To explore the molecular diversity of venom peptide entities in the venom of *H. pingtungensis*, we conducted semi-preparative RP-HPLC purification of the venom and offline MALDI-TOF analysis of eluted fractions. As shown in Figure 4A, the venom is fractionated into 36 fractions in RP-HPLC, for which most fractions are eluted with a retention time between 30 min to 50 min (30–50% acetonitrile gradient). MALDI-TOF MS analysis identified a total of 110 different molecular weights (MWs) in these fractions, with each MW representing a venom peptide [29]. Most venom peptides have an MW between 3500 Da and 4500 Da, which are clustered near the MW of 4000 Da (Figure 4B). We next tested the activities of these fractions on NaV1.7 channels heterologously expressed in HEK293-T cells at a final concentration of 2–3 µM (note, it is an estimated molar concentration which is calculated by dividing the mass concentration of each fraction by its most abundant peptide’s MW, as determined by mass spectrometry analysis). Excitingly, nine fractions showed dramatic inhibition on the NaV1.7 currents (>50% inhibition), with HP-F-24, HP-F-25, HP-F-26, and HP-F-27 exhibiting the most potent activity (approximately 80% inhibition) (Figure 4C, *n* = 5–6). We further purified HP-F-24 and HP-F-25 by analytical RP-HPLC with a much slower acetonitrile gradient and evaluated the inhibitory activity of the finely purified components on the NaV1.7 channel. Finally, two peptide toxins with potent NaV1.7 inhibition were purified to homogeneity, and following the nomenclature rules proposed by King et al. [12], these two toxins were named as µ-Sparatoxin-Hp1 and µ-Sparatoxin-Hp2, respectively (Figure 4D,E). MALDI-TOF MS analysis showed that the MW of µ-Sparatoxin-Hp1 and µ-Sparatoxin-Hp2 is 4884.7012 Da and 4561.9312 Da (M + H^+^), respectively (Figure 4D,E, middle and right panels). Moreover, combining EDMAN degradation and cDNA library analysis revealed the full sequence of these two toxins, with µ-Sparatoxin-Hp1 and µ-Sparatoxin-Hp2 being the cDNA library clones HptTx-208 and HptTx-133, respectively (µ-Sparatoxin-Hp1: H_2_N-ADSGGDAGGDAGADDEGSCKWMFQSCEPPAKCCDGWTCYKGRCNLIL-amide; µ-Sparatoxin-Hp2: H2N-DDDKKECIGHMGWCAWTDGECCEGYRCKLWCRKIIDWL-amide). The identities of these two toxins were also cross-checked by matching their experimentally determined MWs with those derived from their sequences (theoretical MW of µ-Sparatoxin-Hp1 and µ-Sparatoxin-Hp2 is 4883.39 Da and 4560.26 Da, respectively; note that 3 disulfide and the C-terminus amidation in toxin reduces the MW of linear peptide by 7 Da). Furthermore, 3 μM µ-Sparatoxin-Hp1 and µ-Sparatoxin-Hp2 inhibited NaV1.7 currents by 79 ± 1.8% and 80 ± 0.9%, respectively (Figure 4F, *n* = 5–6). These two toxins are novel pioneer molecules for developing analgesics. In summary, these data suggest that *H. pingtungensis* venom contains a great diversity of peptides, which are valuable sources for identifying novel ion channel modulators.

## 3. Conclusions

The present study has uncovered a great diversity of peptide toxin transcripts in the venom gland of *H. pingtungensis* using a combinatorial strategy of cDNA library sequencing and transcriptome sequencing. Moreover, RP-HPLC and MALDI-TOF analysis also confirmed the wealth of peptide entities in the venom, while patch-clamp analysis revealed that many RP-HPLC fractions have potent NaV1.7 channel inhibitory activity and thus, might be valuable for developing analgesics. Interestingly, the sequences variation of peptide toxins in *H. pingtungensis* venom is likely to be much higher than that observed in other spiders in our previous study [11,30,31,32], in which most toxin families contain only 1–2 parental toxin precursors and other toxins could be its/their natural mutants with very few residues mutation. We investigated the abundance of toxins in the three most diverse families (J, L, and M) by countering their transcript’s number in the sequencing data, which revealed that most of them are sequenced by only 1–2 times. It is worth noting that we constructed the *H. pingtungensis* venom gland cDNA library using a normalized cDNA library strategy, which would relatively increase the abundance of low-copy transcripts and consequently facilitate the discovery of these low abundance toxins existing in the venom gland. No reliable phylogenic tree was successfully constructed when we tried to explore the evolvement relationships of all the toxins in the venom gland of *H. pingtungensis*, which suggests that these toxins evolved from several separate toxin ancestors. Finally, most toxins identified in the venom of *H. pingtungensis* are not pharmacologically annotated due to a lack of sequence homology with known toxins and/or lack of hit toxins with an experimentally determined function in the database. Taken together, this study expanded the volume of the spider toxins library and proved that the venom of *H. pingtungensis* is a great starting point for screening ion channels’ modulators.

## 4. Materials and Methods

### 4.1. cDNA Library Sequencing and Transcriptome Sequencing

The spider *H. pingtungensis* (Figure 1A) was captured in Guangxi province in China and maintained in our lab for short time. Approximately 300 mg of venom was collected from about 1000 spiders by an electrical stimulation method. Four days post milking, the venom glands from 5 spiders were dissected and homogenized in liquid nitrogen. Total RNA was extracted, and the cDNA library was constructed using the SMART^®^ cDNA Library Construction kit following the manufacturer’s instructions (Takara Bio USA, Inc., Mountain View, CA, USA). The primary cDNA library was diluted by 10^6^ folds and seeded onto LB (Luria-Bertani) agar plate with ampicillin, cultured overnight at 37 °C, and a single bacteria clone was randomly selected for DNA sequencing using the M13F forward universal primer. Inserted DNA fragment between the designated upstream and downstream sfiI clone sites, with a length of >300 bp, was defined as high-quality EST. Library clones were sequenced until no novel sequence was found. Transcriptome sequencing of the *H. pingtungensis* venom gland was performed in Illumina HiSeq X Ten platform (Illumina, San Diego, CA, USA) in oebiotech (Shanghai OE Biotech. Co., Ltd., Shanghai, China). Briefly, total mRNA was extracted, and the library was constructed using the TruSeq Stranded mRNA LTSample Prep Kit (Illumina, San Diego, CA, USA) according to the manufacturer’s instructions. Short reads were assembled using Trinity [33] (version: 2.4) and the longest transcript was chosen as the unigene, based on similarity and length analysis. Finally, the coding sequence and protein sequence database were constructed by BLAST and ESTscan analysis, and the transcripts encoding the toxin-like peptides were extracted and mixed with ESTs derived from cDNA library sequencing for further analysis.

### 4.2. ESTs Translation and Annotation

The EST sequences were blasted against the non-redundant protein sequence (nr) database using the BLASTx tool (https://blast.ncbi.nlm.nih.gov/Blast.cgi?PROGRAM=blastx&PAGE_TYPE=BlastSearch&LINK_LOC=blasthome; accessed on 23 January 2022); the hit protein (cutoff value is 1 × e^−5^) with the highest score was used to annotate the EST. All ESTs were classified into the following three groups: the toxin-like ESTs which show similarity to known toxins, the cellular component ESTs which matched other cellular proteins rather than toxins, and the no-matched ESTs that did not match any sequences in the database. The corresponding protein sequences database was then made by translating the EST sequences using the EXPASY online translation tool (https://web.expasy.org/translate/; accessed on 23 January 2022). 

### 4.3. ESTs Clustering and Family Analysis of Toxin-like Peptides

ESTs from each group (toxin-like, cellular component and non-matched ESTs) were clustered using the SeqMan Pro application of the DNASTAR Lasergene software (DNASTAR, Inc., Madison, WI, USA) [34]. The toxin-like precursors were grouped into 13 families (families A–M) based on their sequence homology and cysteine frameworks using Cluster [35].

### 4.4. Cell Culture and Transfection 

HEK293-T cells were cultured in Dulbecco’s Modified Eagle Medium (DMEM) (Invitrogen; Thermo Fisher Scientific, Inc., Waltham, MA, USA) supplemented with 10% FBS (Fetal Bovine Serum) and 1% PS(Penicillin-streptomycin) (all from Gibco; Thermo Fisher Scientific, Waltham, MA, USA), in standard conditions (37 °C, 5% CO_2_, saturated humidity). Cells of 80–90% confluence were transfected using Lipofectamine 2000, following the manufacturer’s instructions (Invitrogen; Thermo Fisher Scientific, Inc., Waltham, MA, USA). Briefly, 4 μg human NaV1.7 plasmid plus 0.5 μg pEGFP-N1 plasmid were co-transfected, and 4–6 h later, cells were seeded onto PLL(poly L lysine)-coated coverslips. Then, 24–36 h after transfection, cells were ready for patch-clamp analysis. Positively transfected cells were identified by green fluorescence of EGFP (enhanced green fluorescent protein). 

### 4.5. RP-HPLC Fractionation of H. pingtungensis Venom and Testing the Activity of Venom Components on NaV1.7 Channel

*H. pingtungensis venom* was dissolved in ddH_2_O to a final concentration of 5 mg/mL and fractionated in RP-HPLC (Hanbon Sci. And Tech. Huai’an, China) equipped with a semipreparative C18 column (10 mm × 250 mm, 5 μm; Welch Materials Inc, Shanghai, China), using a 55 min acetonitrile gradient from 5% to 60% at the flow rate of 3 mL/min. For MALDI-TOF MS analysis to reveal the diversity of venom peptides, 1 μL aliquot of each fraction and 1 μL CCA(α-Cyano-4-hydroxycinnamic acid) (20 mg/mL, dissolved in 50% ACN supplemented with 0.1% TFA) was sequentially spotted onto a 96-well target plate, air-dried and analyzed in an AB SCIEX 5800 MALDI-TOF mass spectrometer (AB SCIEX, Foster City, CA, USA). Mass spectra were acquired in a reflectron mode with the following settings: pulse width, 20 ms; vacuum degree, 4 × 10^−7^ torr; acceleration voltage, 25 kV. The mass range was set to 1000 to 10 kDa to identify most of the venom peptides (the matrix peaks are with MW (molecular weight) <1000 Da and venom proteins are with MW > 10 kDa). All the eluted fractions were collected, lyophilized and dissolved in ddH_2_O to make the high-concentration stock solutions; their protein concentration was determined using the BCA quantification kit (Sangon Biotech, Shanghai, China). Fine purification of interested crude fractions was conducted in RP-HPLC (Waters 2795 HPLC system; Waters Corporation, Milford, MA, USA) equipped with an analytic C18 column (4.6 mm × 250 mm, 5 μm; Welch Materials Inc, Shanghai, China) using a 30-min acetonitrile gradient from 30% to 45%, at the flow rate of 1 mL/min. Whole-cell currents recording was performed in an EPC10 USB patch-clamp platform (HEKA Elektronik, Lambrecht, Germany). Pipettes were prepared from glass capillaries using the PC-10 puller (NARISHIGE, Tokoya, Japan). To minimize the pipette capacitance effect, only the tip of the pipette was filled with the pipette solution. The fast and slow capacitance effects were sequentially canceled using the C_f_ and C_m_ compensation function of the amplifier. To minimize the voltage error in the recording circuit, the serial resistance (R_s_) after break-in was kept to be less than 10 MΩ, and 80% Rs compensation with a speed value of 10 µs was used. The pipette solution for recording NaV1.7 currents contains (in mM): 140 CsF, 1 EGTA, 10 NaCl, 10 HEPES (pH = 7.4, adjusted with CsOH); and the corresponding bath solution contains (in mM): 140 NaCl, 5 KCl, 2 CaCl_2_, 1 MgCl_2_·6H_2_O, 10 HEPES, 10 Glucose (pH = 7.4, adjusted with NaOH). Cells were clamped at −80 mV, and NaV1.7 currents were elicited by depolarization to 0 mV. Peptide toxins were applied by using a homemade micro-perfusion system. Briefly, the coverslip with NaV1.7-transfected cells seeded on it was put in the perfusion chamber with 100 μL bath solution, and 100 μL 2-folds concentrated toxin was quickly added using a microsyringe connected with tubing of a small dead volume.

### 4.6. Sequence Determination of µ-Sparatoxin-Hp1 and µ-Sparatoxin-Hp2

The full sequences of µ-Sparatoxin-Hp1 and µ-Sparatoxin-Hp2 were determined by combining EDMAN degradation and cDNA library analysis, as described in our previous studies [28]. Briefly, the N-terminal sequence of the toxin was determined by EDMAN degradation and blasting the resulted sequence against the toxin sequence database as presented in this study, determined the full sequence, and the toxin’s identity was further cross-checked by matching its experimentally determined MW with the theoretical one.

### 4.7. Data Analysis

Electrophysiological data were acquired using PatchMaster software (HEKA Elektronik, Lambrecht, Germany), analyzed using IgoPro 6.10A (WaveMetrics Inc., Portland, OR, USA), Excel 2019 (Microsoft Corporation, Redmond, WA, USA), and Graphpad Prism 9 (GraphPad Software, La Jolla, CA, USA). Data were presented as MEAN ± SD, *n* was presented as the number of separate experimental cells.

## Data Availability

The sequences of peptide toxins reported in this study were deposited in Genbank database with the accession numbers OM362623—OM362812.
